# The American Medical Association

**Published:** 1872-06

**Authors:** 


### THE AMERICAN MEDICAL ASSOCIATION.

The senior editor of this journal had the pleasure of attending the late annual meeting of the National Association. The proceedings were interesting, and we propose in this brief notice to allude to only a few of the most important subjects brought before the body, and to give a short and rapid survey of some of the medical men, colleges, and hospitals we had the gratification of meeting and visiting.

The attendance upon the Association was unusually large, there being about eight hundred delegates present, forming a body of more than ordinary talents, and of high social character; every portion of the Union being represented. The President, Dr. D. W. Yandell, of Louisville, Kentucky, presided. The Association having been opened with prayer by the Right Rev. Bishop Stevens, of Pennsylvania, Professor R. E. Rogers, Chairman of Committee of Reception, delivered an address of welcome, in which he very happily and favorably referred to the triumph of the Faculty of Philadelphia over the diploma venders of the defunct bogus colleges of that city. He spoke eloquently of the common and truly social brotherhood of the medical men of America.

President Yandell's address was an able production, worthy of the heart and head that conceived it. It was pleasantly interspersed with anecdote and humor. He reviewed the early history of medical science in this country, and eulogized Rush, Wistar, and Chapman, the last named having first occupied the Presidential chair of the Association. He argued that the cradle of American liberty should also be the cradle of American medicine. He claims for the Medical men of America equal excellence with those of Europe and every way unwilling to yield to the boasted supremacy of German scientists.

He argued that while practical treatises are needed, that medical science should be learned at the bedside—among the patients of the hospitals—which was acknowledged to be true, and important to be remembered by preceptors and students, if they were indeed in earnest in the desire to raise the standard of medical education.

On the second day Professor R. E. Rogers offered a resolution inviting Dr. Chancellor, of the University of Virginia, to a seat on the floor.

Dr. Bronson offered a resolution that the Committee of Ethics, to consist of seven members, be elected by the Nominating Committee. This resolution, though lost, ought, in our opinion, to have been adopted, and we trust will be at the next annual meeting. We would not by any means be understood as casting any reflection upon the presiding officer or the present committee. We should think it a wise principle to adopt, and the best means of relieving the President of the responsibility, and frequently the unjust reproaches of the disappointed. It would throw a shield around the private character of the good, and be an effective instrument of correcting all infractions of ethics on the part of unruly and scheming members.

Dr. J. S. Weatherly, of Alabama, Chairman of the Committee on Medical Education, presented a report signed by Dr. J. M. Toner and himself, in which was recommended that the Association appeal to the States, asking that no more charters be granted by their Legislatures which do not conform to the plan hereafter to be adopted by the Association; and all colleges now in existence refusing to observe these requirements shall forfeit their charters. The scheme of Dr. Bartlett was recommended as feasible and practicable.

The Committee also urged the establishment of a National Academy of Medicine, as suggested by Dr. Moses, of Missouri; and recommend the expediency of the Association publishing a monthly journal under its auspices, in lieu of the annual volume of Transactions.

Dr. Parvin, Chairman of the Committee on Medical Literature, made a most excellent report. It notices a steady improvement in the character of medical journals. It urges that our medical men should purchase liberally of our medical works. The prize of one hundred dollars, for the best essay, was awarded to Dr. S. R. Percy, of New York. The theme was, "What is the Physiological Value of Phosphorus?"

In the evening the delegates attended a lecture by Dr. H. D. Noyes, on the "Relations of the Disease of the Minor Structure of the Eye to other Affections of the Body," and another by Professor R. E. Rogers, on "Spectral Analysis and the Production of Electricity by Steam."

Handsome and elegant receptions were given at the mansions of Professor Hodge, and Dr. W. H. Pancoast. The refreshments and viands were delicious, just as were expected to be offered by two justly honored representatives of good and distinguished fathers—one of whom, Dr. Pancoast, has been gathered unto his fathers. The other, Dr. Hodge, still lives, and whom we did ourself the honor of visiting. His eyesight has almost failed, but he gave us a cordial greeting, and spoke as kindly and instructively as ever. Long may he live to adorn the profession, and advise and comfort his appreciative family!

On the third day a resolution was offered recommending druggists "to place all external remedies in bottles not only colored so as to appeal to the eye, but also rough upon one side, so that by the sense of touch no mistakes shall be possible in the dark; and that all bottles containing poison should not only be labelled 'poison,' but with another label indicating the most convenient and efficient antidote."

Dr. Stein, of New York, offered a resolution authorizing the appointment of a committee to investigate the transmission of zymodic diseases from animals to human beings, which was adopted.

Dr. F. Horner, of Virginia. offered a resolution to the effect that the members of the association generally, in their practice, will discourage the use of alcoholic stimulants, which was adopted.

D. Warren, Chairman of the Committee on nominations, made a report as follows:

For President, Dr. T. M. Logan, of California.

For Vice Presidents, Drs. B. H. Catlin, of Connecticut; M. C. Pheeters, of Missouri; Pollock, of Pittsburg; and W. T. Briggs, of Tennessee.

For Treasurer, Dr. Casper Wistar, of Philadelphia.

Librarian, Dr. Wm. Lee, of Washington, D. C.

Permanent Secretary, Dr. Wm. B. Atkinson, of Philadelphia.

Assistant Secretary, Dr. Montrose A. Pullen, of St. Louis.

Committee of Library, Dr. J. M. Toner, of Washington, D. C.

Place of next meeting, St. Louis, Missouri.

Dr. N. S. Davis read the report of the Committee on Ethics. The report was averse to the admission of female delegates, and to the members of the Academy of Medicine of Washington, D. C., the Freedmen's Hospital, and the Medical Department of Howard University. It indorsed the recommendations of the Medical Society of Massachusetts in ridding that State of quackery.

During the evening the delegates listened to a well prepared lecture on "Sound," by Dr. J. S. Cohen, of Philadelphia, at the Jefferson Medical College. Dr. Cohen is a rising man. Also on the same evening the delegates were elegantly entertained at the mansion of Col. T. T. Scott, the railroad monarch of America.

Delegates will not soon forget the grand entertainment and the cordial welcome given them by Mr. Scott on that occasion.

Many, also, had an *entree* to the Union League, which is regarded as one of the great novelties of Philadelphia.

On the fourth day resolutions of respect to the late Professor Samuel Jackson, Drs. Gerhard, of Philadelphia, and Pitcher, of Detroit, Michigan, were passed.

Dr. DeCosta was appointed to prepare resolutions in respect to the memory of Professor S. H. Dickson.

Professor Gross recommended that the present system of appointing Standing Committees on Medical Education, Medical Literature, and Climatology and Epidemics, be abolished. His motion for annual lectures upon medicine, surgery, and midwifery, was laid on the table.

Dr. L. Roche offered a resolution inviting the physicians of the world to attend the Medical Congress to be held in 1876, which was lost.

Dr. Skillman, of Kentucky, offered a resolution in which the valuable services of Dr. W. B. Atkinson, Permanent Secretary, was acknowledged, and an annual salary of $500 tendered him.

On motion of Dr. Baldwin, of Alabama, Dr. Sullivan was made chairman of a special committee to consider the relations between the physician and druggist, and to report at the next meeting.

One of the most interesting and attractive features of the preparations made for the entertainment of delegates, was the large number of various and interesting objects placed for inspection in the hall of the College of Physicians.

In the evening the delegates visited Fairmount Park, where they partook of a sumptuous entertainment at the famous Bellemount Pavilion. Speeches were made, toasts offered, and a good time enjoyed.

It is certain the Association never held a more pleasant, harmonious, and satisfactory session.

TO BE CONTINUED.

The proceedings of the Association have been published—since the above was written—by the Secretary, Dr. W. B. Atkinson, which can be had, for fifty cents, by addressing him at Philadelphia, 1400 Pine street.

